# Immunological Sex Differences in Socially Promiscuous African Ground Squirrels

**DOI:** 10.1371/journal.pone.0038524

**Published:** 2012-06-08

**Authors:** Mary Beth Manjerovic, Jane M. Waterman

**Affiliations:** 1 Illinois Natural History Survey/Department of Animal Sciences, University of Illinois, Urbana, Illinois, United States of America; 2 Department of Biological Sciences, University of Manitoba, Winnipeg, Manitoba, Canada; University of Western Ontario, Canada

## Abstract

Differences in how males and females respond to foreign antigens are common across taxa. Such sexual differences in the immune system are predicted to be greater in species with high promiscuity and sociality as these factors increase the likelihood of disease transmission. Intense sperm competition is thought to further this sexual dichotomy as increased investment in spermatogenesis likely incurs additional immunological costs. *Xerus inauris*, a ground squirrel found throughout southern Africa, is extremely social and promiscuous with one of the highest male reproductive investments among rodents. These life-history attributes suggest males and females should demonstrate a large dichotomy in immunity. Contrary to our prediction, we found no difference in spleen mass between the sexes. However, we did find significant biases in leukocyte types and red blood cell counts, possibly reflecting responses to parasite types. Among males, we predicted greater investments in spermatogenesis would result in reduced immunological investments. We found a negative association between testes and spleen size and a positive relationship between testes and number of lice suggesting trade-offs in reproductive investment possibly due to the costs associated with spermatogenesis and immunity. We suggest when measuring sexual differences in immunity it is important to consider the effects of reproductive pressures, parasite types, and life history costs.

## Introduction

Sexual selection imposes different selective pressures on males and females resulting in a dichotomy in fitness strategies [1]. In efforts to maximize their number of progeny, females typically invest more in processes affecting longevity [2], [3]. Male investment is dependent on short term mating success, which can be maximized by increased mating rate often at the expense of immune defenses [3]. Differences in susceptibility to infection may therefore reflect differential selection on males and females as well as sex-biased life-history traits relating to mating and reproduction. Previous research across multiple taxa found tradeoffs between reproduction and immunity that can occur pre- and post-mating, however there is little consistency in the patterns between these two processes when looking at natural populations [1], [4]. This inconsistency may be attributed to the many behavioral and physiological processes an individual utilizes in an effort to counter parasitic infections [5].

Male vertebrates typically show increased susceptibility to disease and higher rates of parasitic infections compared to females [6]–[8]. These differences may be a result of males being in worse body condition [9] but often are attributed to the negative effects of androgens and the positive effects of estrogens[5], [10]–[14]. An individual’s ability to respond to foreign antigens in order to reduce costs associated with infection is often referred to in the literature as ‘immunocompetence’ [15]. Life history trade-offs are such that reproductive strategies often reflect immunological strategies [15], as demonstrated by the robust association between sexual selection and male-biased parasitism [7]. For example, in monogamous species, where pressures to compete for females are less intense, males and females are less likely to differ in disease susceptibility [1], [14]. As competition for mates increases, however, males invest more energy into courtship displays, intrasexual competition, and sperm competition. Such competitive investment is energetically expensive and requires high levels of testosterone to express secondary sex characters [10]. Testosterone is hypothesized to have a dualistic effect, stimulating sexual character development while simultaneously reducing immune response. Folstad & Karter [10] formalized the ‘immunocompetence handicap hypothesis’ (ICHH) based on the assumption that testosterone suppresses immune function during spermatogenesis because sperm are recognized as foreign bodies. Testosterone-dependent characters therefore represent honest indicators of quality because only highly immunocompetent males are capable of trading-off between reproduction and immunity [10], [11]. However, types of parasites may be impacted differently by androgens suggesting this relationship is much more complex than originally thought [16], [17].

The ICHH predicts species with high investment in spermatogenesis are more likely to demonstrate a trade-off between reproductive and immunological investment [10], [18]. This relationship is not always found, possibly because immune response is also dependent on social and environmental circumstances. Likewise, the pattern of lower male immunocompetence compared to females is not always explicit as multiple ecological and behavioral factors play a role in the evolution of immune defenses [3]. Patterns of increased parasitic infection could be a response to differences in habitat quality, food preferences, body size, or sexual differences in life history tactics that directly or indirectly influence parasite burdens [2], [8], [12], [14], [19], [20]. For example, exposure rates due to different mating behaviors or increased home range sizes may cause the sex with greater exposure, often males, to have increased parasitism [7], [14]. Characteristics such as increased sociality and promiscuity increase the likelihood of infection and transmission of pathogens altering immune response [20] and may be different between males and females resulting in variation in parasite loads. In promiscuous systems, individuals have increased contact with others, which increases both the probability of exchanging parasites, particularly ectoparasites, and the risk of acquiring sexually transmitted infections [21], [22].

We addressed sex differences in immunity in the Cape ground squirrel (*Xerus inauris*), a species that is both highly social and promiscuous [23], [24]. Such a high amount of promiscuity results in one of the largest investments in male reproductive anatomy compared to other sciurids [25]. This investment is attributed to males outcompeting others via sperm competition [23], [25]. Although females breed year round [26], estrous opportunities are limited [23], suggesting males are under greater selection to increase short term mating success and therefore invest more in reproductive morphology compared to females who invest in processes affecting longevity and offspring survival. The result of these differing investments is predicted to be lower male immunocompetence compared to females. Previous research supports this prediction with males carry higher ectoparasite loads compared to females, a factor attributed to increased testosterone [27]. Because increases in testosterone are important for sperm production, we predict males that are able to invest more in sperm competition do so at a cost to their immune system resulting in a trade-off between immune and reproductive investment.

## Results

**Figure 1:**
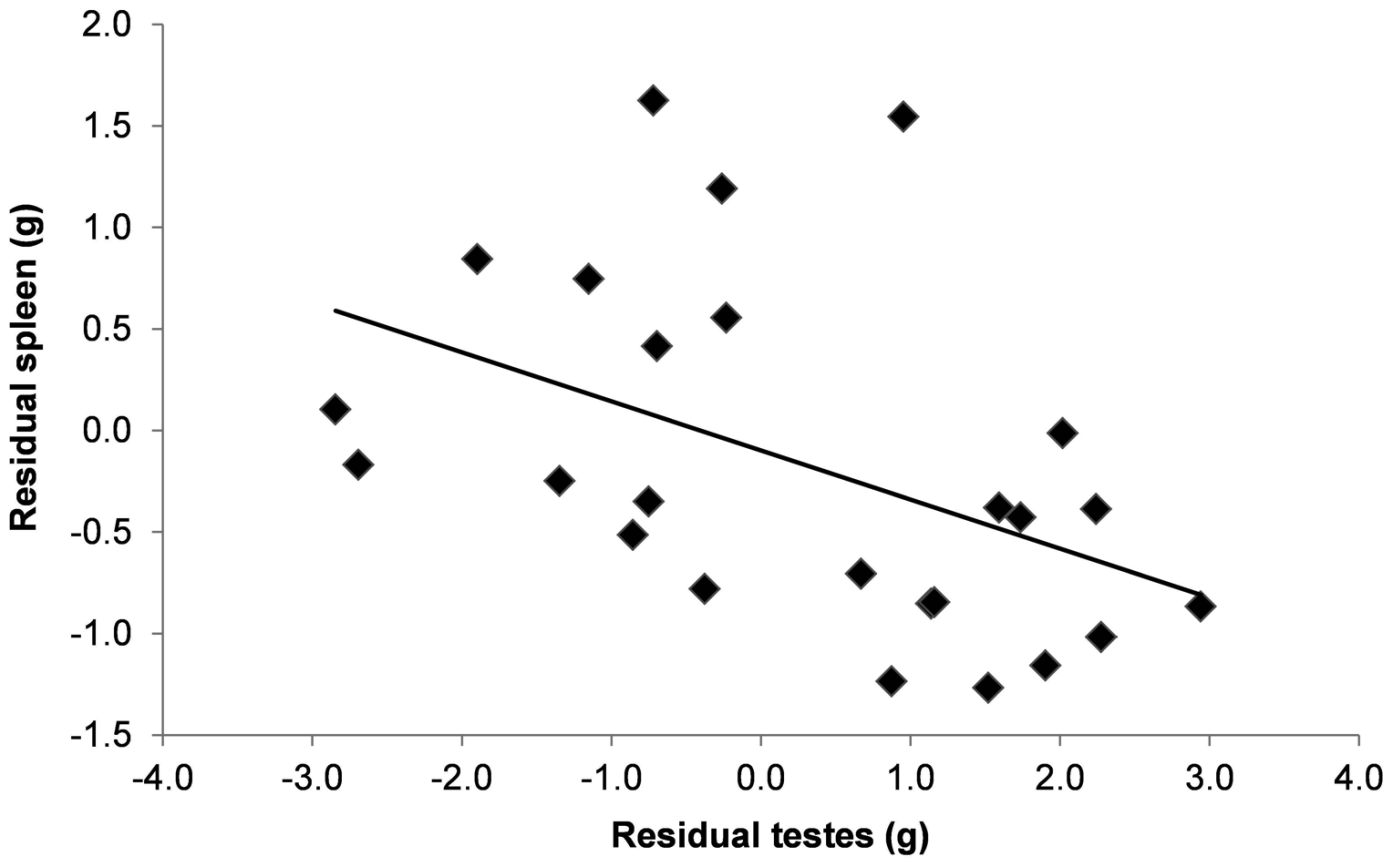
Relationship between residual masses of spleen and testes (*r* ^2^ = 0.21, F_1,23_ = 6.07, *P* = 0.022) calculated from least squares regression against body mass in *Xerus inauris*. Note: two points are overlapping at x = 1.1, y = −0.85.

**Figure 2:**
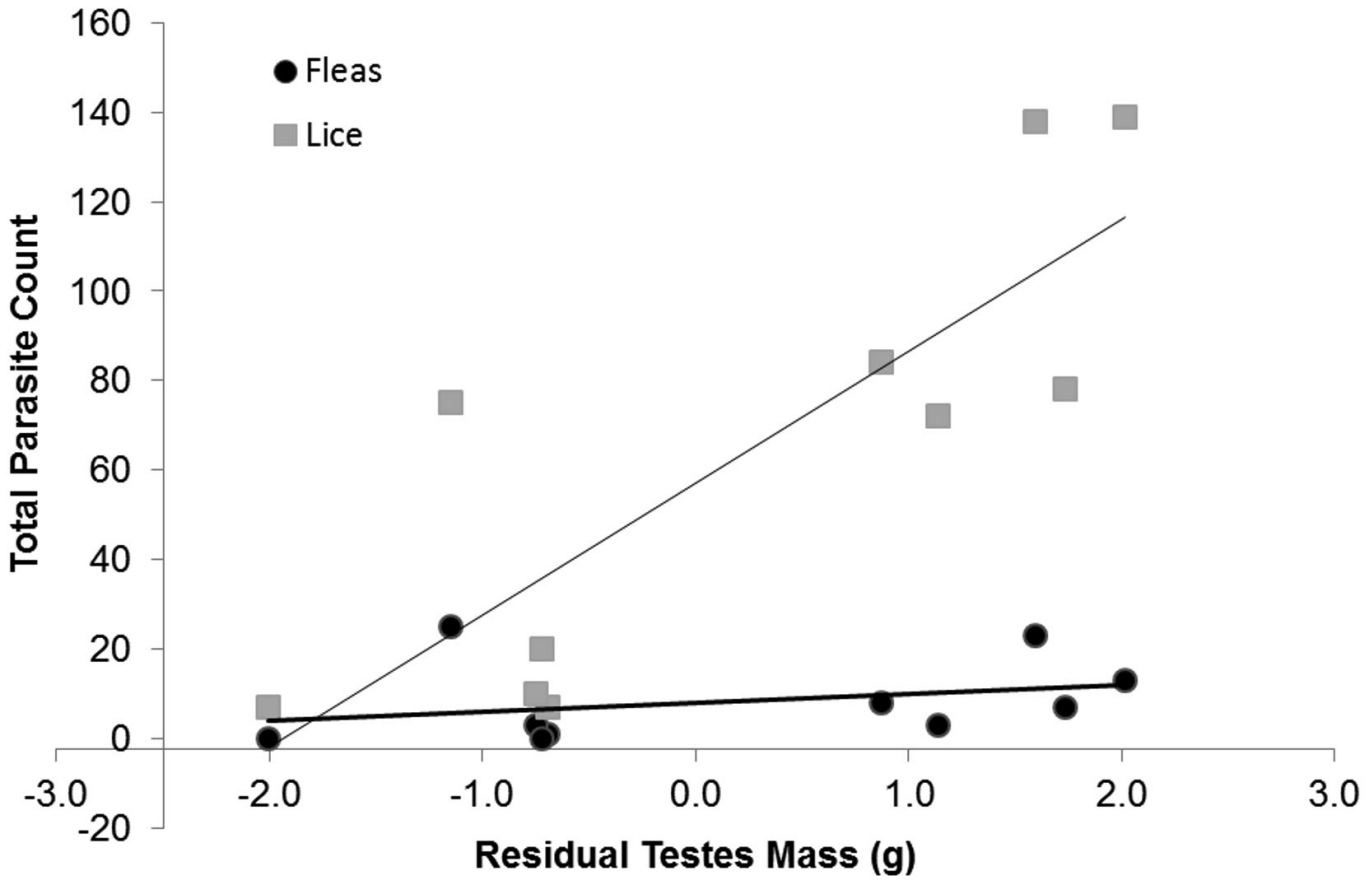
Relationship between residual masses of testes and total flea and lice counts for male *Xerus inauris* (Lice: r^2^ = 0.68, F_1,8_ = 16.97, *P* = 0.003; Fleas: r^2^ = 0.10, F_1,8_ = 0.86, *P* = 0.381).

We euthanized 26 males and 11 females and found no significant differences between body mass (*t*_13.0_ = −0.26, *P* = 0.797) or spleen size (*t*_35_ = −0.26, *P* = 0.800) after controlling for body size. Despite finding no significant difference between body condition of males and females (*t*_10.8_ = 0.26, *P* = 0.798), males did have significantly more ectoparasites per individual (*t*_10_ = 3.19, *P* = 0.005) averaging 71.3±18.3 compared to females (10.8±5.1 per individual). The majority of those ectoparasites for both sexes were lice (males: 63.0±16.1; females: 9.3±5.0). We took blood from live animals in addition to those we euthanized such that we had 7 additional male samples and 22 additional females, although red blood cell counts were not performed on all additional animals. We found the percentage of red blood cells to be significantly lower in males than females (*t*_62_ = 2.97, *P* = 0.004; Table 1). We also found significant differences in white blood cell percentages, with males having higher basophils (*t*_40.2_ = −5.65, *P*<0.001) and lymphocytes (*t*_28.2_ = −2.82, *P* = 0.009) and females having higher percentages of neutrophils (*t*_16.7_ = 2.58, *P* = 0.020) (Table 1). Testes size for one subadult male fit our outlier criterion and was removed. We found a significant negative correlation between the residuals of spleen mass and testes mass (Figure 1). Despite the small sample size, we did find a significant positive correlation between testes mass and lice, after controlling for body mass, but not fleas (Figure 2).

**Table 1:** Average blood cell percentages for male and female *Xerus inauris* (significant differences indicated by bold text).

	Males			Females					
	mean	se	n	mean	se	n	t	df	*P*
**red blood cells**	**45.4**	**2.02**	**27**	**53.3**	**1.10**	**25**	**2.97**	**62.0**	**0.004**
**basophils**	**4.4**	**0.53**	**33**	**1.1**	**0.23**	**33**	**−5.64**	**40.2**	**<0.001**
eosinophils	2.0	0.38	33	1.1	0.35	33	−1.69	31.0	0.102
**neutrophils**	**43.5**	**2.62**	**33**	**56.1**	**3.83**	**33**	**2.58**	**16.7**	**0.020**
**lymphocytes**	**32.2**	**2.29**	**33**	**23.1**	**2.38**	**33**	−**2.82**	**28.2**	**0.009**
monocytes	17.9	1.34	33	18.6	2.16	33	0.26	16.7	0.803

## Discussion

Sex biases in parasitism often are attributed to sex-specific life-history strategies that affect parasite susceptibility and exposure such that differences in reproductive strategies should determine male and female immunocompetence [14]. This rather simplistic view fails to incorporate the ecological and evolutionary dynamics, specifically host-parasite interactions, that may alter male and female immunological response [28]. *X. inauris* have been shown to carry high parasite loads with males having significantly higher ectoparasite loads and females having significantly higher endoparasite loads [27]. Endoparasite variation has been attributed to life history variations between male and female *X. inauris* while ectoparasites are suspected to be under hormonal control [27] suggesting different mechanisms may be employed for countering infections. Contrary to predictions based on these intersexual differences in parasite types and parasite loads, we found no differences in body condition or immunity as measured by spleen size. Spleen mass generally is indicative of overall immune strength [8], [9] and thus similarities in spleen size may be attributed to both sexes being highly infected, albeit with different parasite types [27].

The field of ecological immunity is becoming increasingly complex as variables such as parasite type [29] and host sexual reproduction [28] have been shown to elicit specific immunological responses. Endoparasites and ectoparasites are known to affect hosts differently [30] although rarely are accounted for separately in studies addressing sexual dichotomy of immune response. We found significant differences in percentages of both RBCs and leukocyte types, which may be attributed to variations in parasite type. Lower concentrations of RBCs in males are a likely response to higher numbers of lice and fleas, as they feed directly on host blood and fleas have been shown to induce anemia in rodents [31]. Within the WBC fraction of blood, males have significantly higher percentages of lymphocytes and basophils compared to females, possibly because of higher ectoparasite loads. Basophils are part of an allergic response to ectoparasites [32] while lymphocytes are involved in recognition of antigens and increase in response to inflammation caused by ectoparasites [29], [30]. Conversely, percent of neutrophils were higher in females and have been shown to increase in response to endoparasites [33]. While variation in parasite type has been demonstrated to elicit different immune responses in birds [29], further research is warranted in this species to explicitly link parasite exposure and type to immune response.

Previous studies of immunosuppression imply selection only imposes stress on males during the energetically expensive breeding season [11] and variations in spleen size has been shown to be influenced by sex and stress hormones [34]. *X. inauris*, however, are year-round, asynchronous breeders, requiring both sexes to continuously invest in reproduction [23], [26]. On average, 70% of estruses fail to produce offspring, which has been attributed to costs associated with parasites rather than scarce resources [26], [35], [36]. Parasitic infection often increases when animals reproduce, due to transmission through contact, endocrine changes, and reallocation of resources [37]. Removal of parasites from female *X. inauris* significantly increases reproductive success suggesting females also are immunologically challenged [35]. Given the costs associated with reproduction, females likely face a similar trade-off between investing in reproduction verses immune response. The lack of sexual differences in spleen size suggests immunological strategies in both sexes may be under intense selection in this species.

Significant increases in ectoparasite loads do not arise in males until they reach reproductive maturity [27] suggesting increases in testosterone that accompany reproductive maturity may alter immune response. The ICHH predicts testosterone decreases immunocompetence causing greater vulnerability to infection [10], [38]. Therefore, investing in sexual traits such as testis mass [18] or sperm viability [39] incurs a cost of decreased immune function and represents an evolutionary trade-off if decreased immune function reduces lifespan due to increased infection and mortality. This hypothesis was supported by our data which showed a negative association between testes size and immune function as well as a significant positive association between testes investment and number of lice, despite a low sample size. A similar relationship between testes size and flea and helminth species richness has been found in other rodents which was possibly attributed to testosterone-mediated immunosuppression [40]. However, as our data show, the relationship between testosterone and parasites also depends on the parasite [16], [40], [41]. Similarly, a recent study in spiny lizards (*Sceloporus jarrovi*) demonstrated a positive association between plasma testosterone levels and mites but a negative association with endoparasites [16]. Therefore, male immunity is not universally suppressed by testosterone suggesting these interactions are far more complex than originally thought.

Our findings support the ICHH and the male’s response to testosterone but also suggest a larger issue rarely addressed in the literature. Different parasite types trigger different immune responses. In this system, both males and females have high energetic constraints but significant differences in parasite loads [27]. Therefore, questions involving general immunological stategies measured by a single immune response (e.g. total white blood cells) may fail to detect differences relating to the ecological and evolutionary dynamics of host-parasite interactions and may contribute to mixed support for differences between sexes and the role of hormones in immunity [6]. We suggest future studies on this sexual dichotomy in natural populations should incorporate multiple types of parasites and leukocyte type not just overall numbers of WBCs.

## Materials and Methods

### Ethics Statement

We handled and euthanized animals in accordance with the American Mammal Association guidelines [42] with approval from the University of Central Florida IACUC (#07–43 W).

We sampled *X. inauris* from May - June 2007 at S.A. Lombard Nature Reserve near Bloemhof, South Africa (27°35′S, 35°23′E). We trapped squirrels using live traps (Tomahawk Live Trap Co., Tomahawk, Wisconsin), targeting males for use in multiple studies, and euthanized a subset of adult animals on site with a halothane overdose. While this subset was chosen opportunistically, we are confident that we did not introduce unintentional bias as previous trapping history has no indication of biases in the condition of the animals trapped [43]. We took blood samples from the femoral artery of live, restrained animals or from the internal cavity of euthanized animals. We handled all squirrels in the same manner prior to release or euthanasia thus we do not expect differences in our results based on variation in these methods [44]. We also removed total ectoparasites (fleas and lice) from both males and females.

To assess immunity, we measured spleen size in euthanized animals and percentage of red and white blood cells in all animals. Although such measures are proxies for immunity, they are frequently used to assess immunocompetence [8], [9], [18], [38]. We recorded body mass (±5.0 g) with a spring scale (Pesola AG, Baar, Switzerland) and spleen mass (±0.01 g) on an AccuLab digital scale (Edgewood, NY). To control for differences in body mass, we compared spleen sizes using the residuals of spleen size regressed on body mass. We assessed body condition following methods outlined in Schulte-Hostedde et al. [45]. We calculated residuals of an ordinary least squares regression of spine length (measured from the occipital condyles to the base of the caudal vertebrae) and body mass compared between males and females. We measured percentage of red blood cells (RBCs) by collecting blood in a heparinized capillary tube and spinning for two minutes in a portable microhematocrit centrifuge (International Medical Associates, Inc.). For white blood cells (WBCs), we counted 100 white blood cells on a single layer blood smear stained with eosin nigrosin to obtain a relative differential count of each leukocyte type: basophils, eosinophils, lymphocytes, neutrophils, and monocytes [13], [46]. We normalized all blood cell percentages using an arcsine transformation. All other data were normally distributed. We analyzed data using JMP® v.8.0 (SAS Institute Inc., Cary, NC), comparing males and females for all variables using a t-test and considered results significant if α≤0.05 [47]. We tested equality of variance using Bartlett’s test and reported t prime if variances were found to be unequal (indicated by non-integer degrees of freedom) [47].

To measure male reproductive investment, we measured mass (±0.01 g) of each testis. To remove effects of body size, we calculated the residuals of a least squares regression of total testes on body mass. We removed outliers if the value fell two standard errors outside of the mean. We then compared relationships between residuals (spleen versus total testes) using a least squares regression [18]. We used the residuals of testes size and body mass in a regression on total ectoparasite loads, which we obtained for a subset of male squirrels (n = 10).
